# GP's Adherence to Guidelines for Cardiovascular Disease among Elderly: A Quality Development Study

**DOI:** 10.1100/2012/767892

**Published:** 2012-05-03

**Authors:** Sara Modig, Peter Höglund, Margareta Troein, Patrik Midlöv

**Affiliations:** ^1^Division of Family Medicine, Department of Clinical Sciences in Malmö, Skåne University Hospital, Lund University, 205 02 Malmö, Sweden; ^2^Tåbelund Primary Health Care Centre, Solv. 33, 241 31 Eslöv, Sweden; ^3^RSKC Competence Centre for Clinical Research, Skåne University Hospital, 22 185 Lund, Sweden

## Abstract

*Background*. Evidence-based guidelines should in most cases be followed also in the treatment of elderly. Older people are often suboptimally treated with the recommended drugs. *Objectives*. To describe how well general practitioners adhere to current guidelines in the treatment of elderly with cardiovascular disease and evaluate local education as a tool for improvement. *Method*. Data was collected from the medical records of patients aged ≥65, who visited a primary health care center in Sweden 2006 and had one or more of the following diagnoses: hypertension, ischemic heart disease, heart failure, chronic atrial fibrillation, or prior stroke. Local education was organized and included feed-back to the patient's doctor and discussion about regional guidelines. Repeated measurements were performed in 2008. *Results and Conclusion*. The adherence to guidelines was low. Approximately one-third of the patients with hypertension reached target blood pressure, stroke patients more often. More patients with heart failure were treated with angiotensin converting enzyme inhibitor than in other European countries, but still only 60%. Half of the patients with chronic atrial fibrillation were treated with Warfarin, although more than two-thirds had a CHADS_2_ score indicating the need. Educational efforts appeared to increase the adherence and hence should be encouraged.

## 1. Introduction

Many elderly are treated with several different drugs. With an increase in the number of medicines, there is a greater risk of interactions and adverse effects. There are however also risks associated with the underuse of recommended treatment. For several diseases, it has been shown that it is more common for elderly than for younger patients to receive suboptimal treatment with the recommended drugs [1–3]. Regional guidelines for the treatment of cardiovascular diseases, built on evidence based data, should in most cases be followed also in the diagnosis and treatment of elderly [4].

*Hypertension* is a well-documented risk factor for cardiovascular disease. There is strong evidence that elderly benefit from antihypertensive treatment at least as much as younger do, probably due to higher risk for cardiovascular events [5]. The recommendation is a target blood pressure (BP) of 130/80 for patients with diabetes and of 140/90 for others [4]. However, recent research has shown that lower systolic blood pressure (SBP) seems to be associated with greater mortality in patients aged 85 or more, irrespective of health status. An optimal SBP for this age group could be above 140 mmHg [6].

The recommendation for *ischemic heart disease* is treatment with aspirin, beta-blocker, and, if the cholesterol level is above recommended, also statin [4]. There is evidence for lowered mortality and morbidity using such treatment, even if only a small number of patients in studies about coronary disease are more than 75 years old.

For the diagnosis *heart failure*, cardiac dysfunction has to be objectively confirmed. The recommended method is echocardiography (ECO). Recommended pharmacological therapy for heart failure is angiotensin converting enzyme inhibitors (ACEIs) which have a well-established effect on mortality and morbidity [4, 7] and for NYHA (New York Heart Association) functional class II-IV also beta-blockers. NYHA functional classification grades the severity of heart failure symptoms. Unfortunately ACEIs are often underused for older patients and especially in nursing homes [2]. Diuretics are overused instead.

For patients with *chronic atrial fibrillation*, anticoagulation should be given to lower the risk of stroke [4]. This risk is depending on age and comorbidity and can be calculated with the CHADS_2_ score, giving one point each for the presence of congestive heart failure, hypertension, age 75 years or older, and diabetes mellitus, and two points for history of stroke or transient ischemic attack (TIA) [8]. Warfarin is indicated at a score of two points or more. Although warfarin has the most positive effect in the elderly, only half of the patients in this age group are treated with warfarin, compared to 85% in the group 45–65 years [9]. If warfarin is contraindicated, aspirin in the dose 320 mg per day could be considered according to the guidelines 2006 [4].

Aspirin is also the best documented secondary prevention of *stroke* [10] in combination with lowering the blood pressure to target 140/90 [4].

This study was performed as a quality improvement project on a middle-sized family health care centre. We wanted to describe how well general practitioners (GPs) adhere to current guidelines when they investigate and treat elderly patients with cardiovascular disease. We also wanted to evaluate feedback and local continuing education as an act for improvement of this treatment.

## 2. Methods

The study was performed in 2006–2008 in a family health care center in Eslöv, a municipality in southern Sweden with a population of approximately 30 000. The center has a registered population of 10500. All nine GPs at the center participated in the intervention. Data was collected from the patient's medical records on patients who met the following inclusion criteria:

- ≥65 years,
- visited the health care center between April (July for hypertension) and December 2006,
- on this visit, received one or more of the following diagnoses: hypertension, heart failure (HF), ischemic heart disease (IHD), chronic atrial fibrillation (CAF), or prior stroke (inclusive TIA).

For the diagnosis hypertension, patients who received the diagnosis less than 6 months ago were excluded.

### 2.1. Measurements and Procedure

The medical records were systematically reviewed by a doctor and the following variables were investigated, as recommended in the guidelines of 2006.

For *hypertension*:

- Target blood pressure reached (130/80 for diabetes and 140/90 for others)?
- If not, number of antihypertensive drugs?
- ECG during the last 2 years?
- Blood lipids tested during the last 5 years?

For *ischemic heart disease*:

- Diagnosis based on cardiac stress test/myocardial scintigraphy/prior verified acute myocardial infarction?
- Treated with betablocker?
- Treated with aspirin (or clopidogrel/warfarin)?
- Blood lipids tested during the last 5 years?
- Target level for cholesterol reached (LDL ≤ 2.5 mmol/L and total cholesterol ≤ 4.5 mmol/L)?

For *heart failure*:

- Diagnostics performed: Echocardiography performed ever? NT-proBNP tested ever? Pulmonary X-ray performed initially?
- Treated with ACEI or, if intolerant, angiotensin receptor blocker (ARB)?
- Target dose used for ACEI/ARB?
- Treated with betablocker with indication for HF?
- Treated with NSAID regularly?

For *chronic atrial fibrillation*:

- Treated with warfarin? If no, treated with aspirin 320 mg?
- Which CHADS_2_-score does the patient reach?

For *stroke*:

- Target blood pressure 140/90 reached?
- If ischemic stroke: Treated with recommended anticoagulation (aspirin 160 mg, warfarin or clopidogrel)?

Any information not easily accessible in the medical records was regarded as missing. Only a very small number of patients (<10) were excluded due to insufficient quality of data. Patients with hypertension were divided into one group with patients aged 65–84 and one group aged 85 or more. This was due to the recent research finding that the oldest elderly perhaps do not gain from a blood pressure treated to the same low target as younger adults [6].

### 2.2. Intervention

If current recommendations were not followed, feed-back was given to the patient's doctor with suggestions for possible improvements. Local education was organized in 2007 for all clinicians at the health center, focusing on cases and the regional guidelines. The education was repeated and followed up after three months. Repeated measurements were performed for the patients who visited the health care center between April and December 2008.

### 2.3. Statistics

Power calculations for changes in proportions of adherence to guidelines were performed before the study started. For each diagnosis one variable was considered as the main variable. These were for hypertension the proportion who reached target blood pressure, for IHD treatment with beta blocker, for heart failure treatment with ACEI/ARB, for CAF treatment with warfarin, and for stroke reaching target blood pressure. The proportion of the main variable was analyzed in a smaller sample. The power calculation was performed on clinically reasonable improvements in the variables. For example, the proportion who reached target blood pressure in the sample was 35% and a clinically relevant proportion in the hypertension population should be 60%. The observation period was set up in order to get sufficient number of patients. It was enough to investigate a random sample of every fifth patient with hypertension aged 65–84. For IHD, HF, and CAF adequate numbers of patients were available during the observation period. Enough stroke patients were not found at the center to expect significant changes. Observed proportions of adherence and their exact confidence limits were calculated. The proportions from the studied periods were compared using two-sample test for equality of proportions. Computer software R version 2.6.0 was used for all statistical analyses (R Foundation for Statistical Computing, Vienna, Austria).

### 2.4. Ethics

The study did not require an ethical approval according to a protocol by the ethical committee of Lund University no. 228/2007. The study was performed in accordance with Swedish law and the declaration of Helsinki.

## 3. Results

Characteristics of the patients with hypertension are presented in Table 1. Approximately one-third reached target BP. Of those who did not reach target BP and were aged 65–84, 29% (8/28) had monotherapy in 2006 and 34% (12/35) in 2008. Of those aged 85 or more and who did not reach target BP, 53% (8/15) had monotherapy in 2006 and 36% (10/28) in 2008.

The measurements of 2006 and 2008 of the patients with ischemic heart disease are presented in Table 2. The treatment with beta blocker and especially aspirin was stable and high.

For patients with HF, treatment with beta blocker increased. Characteristics of HF patients are shown in Table 3. Of these patients 76% (2006) and 88% (2008), respectively, had been examined with pulmonary X-ray as a part of the investigations for diagnosis. No patient with heart failure was treated with NSAIDs regularly in 2006 and one patient was in 2008. Of the patients treated with ACEI/ARB sixteen out of forty-five (36%) were treated up to target dose in 2006 and twenty-three out of sixty-seven (34%) in 2008.

The measurements in 2006 and in 2008 of the patients with chronic atrial fibrillation or prior stroke are presented in Table 4. For stroke, the difference between 2008 and 2006 is not presented, since there were too few stroke patients at the center to expect significant changes. Approximately one-third of the stroke patients reached target BP. About half of the CAF patients were treated with warfarin. Patients with lower CHADS_2_-score were often treated with warfarin.  Table 5 presents the number of CAF patients with different CHADS_2_-score, who are treated with Warfarin, aspirin 320 mg, or no recommended anticoagulation, respectively.

## 4. Discussion

As previously described in other studies in primary care, the adherence to guidelines for the treatment of cardiovascular disease was low in this study. In addition, the study revealed that educational efforts may have a positive impact on the adherence and therefore should be encouraged.

The study was performed as a quality improvement project on a family health care station. We applied the regional guidelines of 2006. These are built on evidence-based data and widely known guidelines of international societies.

In 2006, the recommendation for the treatment of hypertension was a target BP of 130/80 for patients with diabetes and 140/90 for others. For patients aged between 65 and 80, we found a trend of more patients reaching target level after the education intervention (28% in 2006 versus 35% in 2008) but this was not significant (*P* = 0.63). For patients aged above 80 however, the trend was rather in the opposite direction. The modest amount of values reaching target level is in agreement with a prior study within primary healthcare in southern Sweden in 2006, where only 20% of the treated hypertensives reached target level of BP [11]. In that study, 40% of the patients had monotherapy, although combination treatment is recommended. This is in agreement with our findings: one-third of the patients not reaching target level did not have combination treatment in 2008. Another Swedish study also found that old age of the patient seemed to be an important barrier among GPs when considering pharmacological treatment for the management of hypertension [12].

The Hypertension in the Very Elderly Trial (HYVET) revealed that using antihypertensive drug therapy decreases the risk of new cardiovascular events as well as total mortality in the elderly (over 80) [13]. The HYVET study, however, does not provide data on a target BP for these elderly. Molander et al. found that an SBP level of 162 was associated with the lowest mortality in the patient group aged above 85 [6]. The question arises whether many GPs already consider this BP level ideal for this elderly group. HYVET subjects were generally rather healthy. Therefore, it is important to note that results cannot be extrapolated to the frail elderly [14]. GPs might restrict the blood pressure treatment when patients have extensive comorbidity.

Treating blood pressure to target is important in secondary prevention of stroke. The HYVET study showed a 30% reduction in strokes in the treatment group [13]. Therefore the proportion of patients with prior stroke reaching target BP 140/90 should be higher in our study. The proportion was 62% (2006) and 68% (2008), respectively. The physicians seemed more eager to treat to target in the population with prior stroke, than in the population with only hypertension. Another Swedish study showed that the incidence of first-ever stroke was strongly related to poor BP control. It was estimated that 45% to 52% of all strokes among pharmacological-treated patients were attributable to uncontrolled blood pressure [15].

In the patient group over 80 with hypertension, we found a significant improvement from 2006 to 2008 in testing of the patient's lipid level. This is not the most important part of the intervention but it reveals that the educational program has an effect.

The positive trend of increased lipid level testing, as well as treatment to lipid target level, was also seen among the patients with IHD. For these patients, we also found a positive trend for the treatment with beta-blocker, from 62% in 2006 to 72% in 2008 (not significant). This is more than was seen in Finland 2003, where 51% of patients with IHD aged 75 and above were treated with beta-blocker [16]. Aspirin treatment or other anticoagulation was in our study stable and high in 2006 as well as in 2008.

Remme et al. found that despite the widespread availability of evidence-based guidelines, there are differences between physicians and countries in the management of HF [17]. HF is objectively diagnosed by ECO, which is recommended in Swedish guidelines. In our study, 56% of the patients with HF had been examined with ECO at some point in 2006. This was increased to 66% (*P* = 0.24) in 2008 after the intervention. There might be room for even further improvement, since 75% of Swedish GPs considered ECO necessary for the diagnosis, according to Remme et al. [17]. We also found a significant improvement in the testing of NT-proBNP, from 43% to 89%, between 2006 and 2008. Perhaps the test has become more frequently used in general these years, but the effect of the educational program should not be underestimated.

Only 43% of European GPs would always, and 51% often, prescribe an ACEI when treating an HF patient. Correspondingly, 59% of the Swedish GPs often, and 34% always, prescribe ACEI. This is similar to the results from our study where 60% of the HF patients were treated with ACEI in 2006. The proportion was 71% in 2008. The increase is not significant (*P* = 0.17) but is perhaps a positive trend. Approximately 35% were treated to target dose, both in 2006 and 2008. Larger proportions cannot be expected, since this patient group is drug sensitive due to age. Many patients have renal impairment, which motivate lower target dose.

The use of beta blockade for HF treatment has increased significantly from 29% to 49% (*P* = 0.015) between our measure times. According to Remme et al. 65% of Swedish GPs would add a beta-blocker in a patient who does not improve despite optimal treatment with a diuretic and an ACEI. Forty-three percent often prescribe beta blockade to HF patients [17]. This might indicate an increasing awareness of the guidelines in Sweden and the improvement in our study may not only be due to the intervention.

Almost no patient with HF was prescribed NSAID regularly, which also indicates an awareness of the recommendations.

Chronic atrial fibrillation is common among the elderly in primary health care, and about half of these patients are treated with warfarin, according to a Swedish study from 2004 [18]. This is close to our findings, where 44% were treated with Warfarin in 2006 and 51% in 2008. According to the findings when using CHADS_2_, the numbers treated with Warfarin should be higher, since 70% of the patients had a score of 2 points or more 2008 (68% 2006). With only one risk factor and CAF, the physician could consider aspirin as anticoagulation. When this study started, the recommendation was aspirin in the dose 320 mg for CAF. We found a significant increase in the use of the recommended dose between 2006 and 2008 (12% versus 39%, *P* = 0.01), which is probably an effect of the intervention. In the guidelines of 2011, an aspirin dose of 75 mg is suggested. There is no evidence-based data that confirms the optimal dose. However, in the cases where aspirin could be considered (score 0-1), no patient out of 22 was treated with aspirin 320 mg in 2008. Fourteen out of the fifty-one with higher scores were treated with aspirin. Perhaps we traditionally select the wrong patients for anticoagulation.

There is still much to improve regarding compliance to guidelines. More local education, as in this project, could be one way. GPs attitudes towards guidelines are yet another factor to deal with. A Swedish study about attitudes revealed that the degree of reliance on research data varied among GPs. Some were convinced of an actual and predictable risk for the individual; others strongly doubted it. Some were relying firmly on protection from disease by pharmaceutical treatment; others were strongly questioning its effectiveness in individual cases [19]. A Croatian study among GPs and internists showed that many primary care physicians use their own personal experience in prevention while internists and cardiologists show a larger tendency to use the guidelines [20].

Hence, since we can show an effect of education programs like the present one, we should encourage a more widespread use of them. Still we cannot be definitely sure of the effect due to the intervention, since some of the positive results could be explained by an increasing awareness in the medical community about investigations or some of the treatments.

More research is needed about optimal treatment, doses, and targets for these elderly, in order to make the guidelines more specific for this population. This would presumably also increase GPs' adherence.

This study has some limitations. Firstly we did not have a control group that was not exposed to the intervention. This means that we cannot eliminate other influences on the results, such as better availability of laboratory and other tests or wishes of the GPs to conform to local quality indicators. However, the project is not first and foremost an intervention study, but a description of how it is possible to work with quality improvement in the clinics and on small units. Second, we identified the patients through diagnoses in the medical records. There might be patients who were not captured, but there is no reason to believe that they differ from the investigated patients.

## 5. Conclusion

By showing that adherence to guidelines about the treatment of cardiovascular disease is low in primary care, we confirm the results of previous studies. For example, only approximately one-third of the patients with hypertension reach target blood pressure. One half of the patients with CAF are treated with Warfarin, although more than two-thirds have a CHADS_2_ score of 2 or more. Educational efforts appear to increase the adherence and therefore should be encouraged. For example, the use of beta blockade to heart failure patients and the assessment of NT-proBNP in these patients increased significantly after the intervention.

## Figures and Tables

**Table 1:** Patients with hypertension who reach target blood pressure and are investigated with ECG and blood lipids.

		*N*	Percentage (95% CI)	Difference^a ^ (95% CI)	*P* value
Patients 65–84 years					
Total number of patients	2006	39			
	2008	54			
					
Target blood pressure level	2006	11	28.2 (15.0, 44.9)	7.0 (−14.2, 28.2)	0.63
	2008	19	35.2 (22.7, 49.4)		
					
ECG taken last two years	2006	17	43.6 (27.8, 60.4)	−4.7 (−27.2, 17.8)	0.81
	2008	21	38.9 (25.9, 53.1)		
					
Lipids assessed	2006	29	74.4 (57.9, 87.0)	1.6 (−17.8, 21.0)	1
	2008	41	75.9 (62.4, 86.5)		

Patients ≥85 years					
Total number of patients	2006	24		
	2008	41		
					
Target blood pressure level	2006	9	37.5 (18.8, 59.4)	−5.8 (−33.1, 21.6)	0.31
	2008	13	31.7 (18.1, 48.1)		
					
ECG taken last two years	2006	14	58.3 (36.6, 77.9)	−7.1 (−35.4, 21.2)	0.77
	2008	21	51.2 (35.1, 67.1)		
					
Lipids assessed	2006	3	12.5 (2.7, 32.4)	26.5 (3.3, 49.8)	0.047
	2008	16	39.0 (24.2, 55.5)		

^a^Difference between 2008 and 2006.

**Table 2:** Patients with ischemic heart disease who are pharmacologically treated and investigated according to the guidelines.

		*N*	Percentage (95% CI)	Difference^a^ (95% CI)	*P* value
Number of patients	2006	113			
	2008	105			

Investigation performed	2006	105	92.9 (86.5, 96.9)	0.54 (−8.0, 6.9)	1
	2008	97	92.4 (85.5, 96.7)		

Treated with beta blocker	2006	70	61.9 (52.3, 70.9)	10.5 (−2.9,23.7)	0.14
	2008	76	72.4 (62.8, 80.7)		

Treated with aspirin	2006	100	88.5 (81.1, 93.7)	−1.8 (−11.5, 7.9)	0.84
	2008	91	86.7 (78.6, 92.5)		

Treated with aspirin/warfarin	2006	107	94.7 (88.8, 98.0)	−1.4 (−8.6, 5.9)	0.89
	2008	98	93.3 (86.7, 97.3)		

Lipids assessed	2006	84	74.3 (65.3, 82.1)	6.6 (−5.3, 18.5)	0.31
	2008	85	81.0 (72.1, 88.0)		

Target lipid level	2006	32/84	38.1 (27.7, 49.3)	9.0 (−7.1, 25.0)	0.31
	2008	40/85	47.1 (36.1, 58.2)		

^a^Difference between 2008 and 2006.

**Table 3:** Patients with chronic heart failure who are pharmacologically treated and investigated according to the guidelines.

		*N*	Percentage (95% CI)	Difference^a^ (95% CI)	*P* value
Number of patients	2006	75			
	2008	94			

Investigation with ECO	2006	42	56.0 (44.1, 67.5)	10.0 (−6.0, 25.9)	0.24
	2008	62	66.0 (55.5, 75.4)		

Investigation with plasma NT-proBNP	2006	32	42.7 (31.3, 54.6)	46.7 (32.7, 60.7)	<10^−4^
	2008	84	89.4 (81.3, 94.8)		

Treated with ACEI or ARB	2006	45	60.0 (48.0, 71.1)	11.3 (−4.30, 26.9)	0.17
	2008	67	71.3 (61.0, 80.1)		

ACEI recommended dose	2006	16/45	35.6 (21.9, 51.2)	−1.3 (−20.4, 18.0)	1
	2008	23/67	34.3 (23.2, 46.9)		

Treated with beta-blocker	2006	22	29.3 (19.4, 41.0)	19.6 (4.0, 35.2)	0.015
	2008	46	48.9 (38.5, 59.5)		

^a^Difference between 2008 and 2006.

**Table 4:** Patients with chronic atrial fibrillation or prior stroke who are treated according to the guidelines.

		*N*	Percentage (95% CI)	Difference^a^ (95% CI)	*P* value
Chronic atrial fibrillation					
Treated withwarfarin	2006	34/77	44.2 (32.8, 55.9)	6.5 (−10.8, 23.8)	0.52
	2008	37/73	50.7 (38.7, 62.6)		
					
Treated with aspirin 320 mg if not warfarin	2006	5/43	11.6 (3.9, 25.1)	27.3 (6.1, 48.4)	0.010
	2008	14/36	38.9 (23.1, 56.5)		

Stroke					
Target blood pressure level	2006	31/50^b^	62.0 (47.2,75.3)		
	2008	40/59^b^	67.8 (54.4,79.4)		
					
Treated with recommended anticoagulation	2006	35/48^b^	72.9 (58.2,84.7)		
	2008	45/57^b^	78.9 (66.1,88.6)		

^a^Difference between 2008 and 2006.

^b^Two patients had a bleeding stroke.

**Table 5:** CHADS_2_ score of patients with CAF, related to anticoagulation. The table shows the number of patients who are treated with Warfarin, Aspirin, or no recommended anticoagulation, respectively.

CHADS_2_ score 2006	N	Warfarin	Aspirin 320 mg	No anticoagulation
0	5	2	0	3
1	20	13	0	7
2	22	11	2	9
3	12	1	2	9
4	13	4	1	8
5	5	3	0	2
6	0	0	0	0

Total number	77	34	5	38

CHADS_2_ score 2008				

0	5	4	0	1
1	17	11	0	6
2	25	9	8	8
3	18	7	6	5
4	6	5	0	1
5	2	1	0	1
6	0	0	0	0

Total number	73	37	14	22

## References

[1] Ramsay SE, Whincup PH, Lawlor DA (2006). Secondary prevention of coronary heart disease in older patients after the national service framework: population based study. *British Medical Journal*.

[2] Litaker JR, Chou JY (2003). Patterns of pharmacologic treatment of congestive heart failure in elderly nursing home residents and related issues: a review of the literature. *Clinical Therapeutics*.

[3] Klarin I, Fastbom J, Wimo A (2003). A population-based study of drug use in the very old living in a rural district of Sweden, with focus on cardiovascular drug consumption: comparison with an urban cohort. *Pharmacoepidemiology and Drug Safety*.

[4] Läkemedelsrådet Region Skåne (2006). *Background to recommendations of therapeutic alternatives in Skåne*.

[5] Scott AK (2004). Hypertension in older adults. *Reviews in Clinical Gerontology*.

[6] Molander L, Lövheim H, Norman T, Nordström P, Gustafson Y (2008). Lower systolic blood pressure is associated with greater mortality in people aged 85 and older. *Journal of the American Geriatrics Society*.

[7] Remme WJ, Swedberg K (2002). Comprehensive guidelines for the diagnosis and treatment of chronic heart failure: task force for the diagnosis and treatment of chronic heart failure of the European Society of Cardiology. *European Journal of Heart Failure*.

[8] Gage BF, Waterman AD, Shannon W, Boechler M, Rich MW, Radford MJ (2001). Validation of clinical classification schemes for predicting stroke: results from the National Registry of Atrial Fibrillation. *Journal of the American Medical Association*.

[9] Vasishta S, Toor F, Johansen A, Hasan M (2001). Stroke prevention in atrial fibrillation: physicians’ attitudes to anticoagulation in older people. *Archives of Gerontology and Geriatrics*.

[10] Baigent C, Sudlow C, Collins R, Peto R (2002). Collaborative meta-analysis of randomised trials of antiplatelet therapy for prevention of death, myocardial infarction, and stroke in high risk patients. *British Medical Journal*.

[11] Hedblad B, Nerbrand C, Ekesbo R (2006). High blood pressure despite treatment: results from a cross-sectional primary healthcare-based study in southern Sweden. *Scandinavian Journal of Primary Health Care*.

[12] Midlöv P, Ekesbo R, Johansson L (2008). Barriers to adherence to hypertension guidelines among GPs in southern Sweden: a survey. *Scandinavian Journal of Primary Health Care*.

[13] Beckett NS, Peters R, Fletcher AE (2008). Treatment of hypertension in patients 80 years of age or older. *New England Journal of Medicine*.

[14] Kithas PA, Supiano MA (2010). Practical recommendations for treatment of hypertension in older patients. *Vascular Health and Risk Management*.

[15] Li C, Engstrom G, Hedblad B, Berglund G, Janzon L (2005). Blood pressure control and risk of stroke: a population-based prospective cohort study. *Stroke*.

[16] Hiitola PK, Enlund H, Sulkava RO, Hartikainen SA (2007). Changes in the use of cardiovascular medicines in the elderly aged 75 years or older—a population-based Kuopio 75+ study. *Journal of Clinical Pharmacy and Therapeutics*.

[17] Remme WJ, McMurray JJV, Hobbs FDR (2008). Awareness and perception of heart failure among European cardiologists, internists, geriatricians, and primary care physicians. *European Heart Journal*.

[18] Nilsson GH, Björholt I (2004). Occurrence and quality of anticoagulant treatment of chronic atrial fibrillation in primary health care in Sweden: a retrospective study on electronic patient records. *BMC Clinical Pharmacology*.

[19] Silwer L, Wahlström R, Lundborg CS (2010). Views on primary prevention of cardiovascular disease-an interview study with Swedish GPs. *BMC Family Practice*.

[20] Reiner Ž, Sonicki Z, Tedeschi-Reiner E (2010). Physicians’ perception, knowledge and awareness of cardiovascular risk factors and adherence to prevention guidelines: the PERCRO-DOC survey. *Atherosclerosis*.

